# Two-point calibration protocol for the Förster Resonance Energy Transfer indicator Pyronic in neurons

**DOI:** 10.1117/1.NPh.12.S2.S22807

**Published:** 2025-10-22

**Authors:** Felipe Baeza-Lehnert, Yasna Contreras-Baeza, Camila Aburto, Alejandro San Martín

**Affiliations:** a Leipzig University, Carl-Ludwig-Institute of Physiology, Faculty of Medicine, Leipzig, Germany; b Centro de Estudios Científicos (CECs), Valdivia, Chile; c Universidad San Sebastián, Facultad de Medicina, Valdivia, Chile

**Keywords:** pyruvate, Pyronic, fluorescence, metabolism, calibration, neurons, quantification

## Abstract

**Significance:**

Pyruvate is a nodal intermediate in cellular metabolism, positioned at the crossroads between glycolysis and fermentative metabolism. It is exchanged between the intracellular and extracellular compartments through the proton-coupled monocarboxylate transporters and between the cytosol and mitochondria through the mitochondrial pyruvate carrier, where it serves as a primary carbon source for respiration.

**Aim:**

Our goal is to present a detailed protocol for quantifying cytosolic pyruvate concentration in neurons at single-cell resolution using a minimally invasive, two-point calibration approach with the Förster Resonance Energy Transfer (FRET)-based genetically encoded fluorescent indicator Pyronic.

**Approach:**

This protocol is based on a noninvasive pharmacological two-point calibration approach, where Pyronic’s dynamic range ($\Delta R_{\mathrm{MAX}}$) is established by withdrawing all extracellular substrates to deplete intracellular pyruvate ($R_{\mathrm{MIN}}$) and by inducing Pyronic saturation ($R_{\mathrm{MAX}}$) through the combination of inhibition of pyruvate export, stimulation of its production, and blockade of its mitochondrial consumption. The protocol also incorporates the previously published $K_{D}$ values for Pyronic obtained from *in vitro* experiments. This procedure does not require the use of detergents to permeabilize the cells.

**Results:**

Implementing this protocol enables the measurement of absolute cytosolic pyruvate concentrations. This quantitative parameter facilitates comparisons of pyruvate metabolism across different cells, samples, and experimental batches, thereby enabling the comparison between a plethora of experimental conditions.

**Conclusion:**

The FRET-based fluorescent indicator Pyronic can be reliably calibrated using a minimally invasive, pharmacology-based two-point calibration protocol in neurons, thus providing a robust and quantitative method to study pyruvate metabolism under various physiological and pathological scenarios.

## 1 Introduction

Metabolism has recently gained renewed attention since metabolic dysfunction has been linked to the development of several pathologies, especially in the aging brain, where glycolytic dysfunction has been detected.^1^^,^^2^ Pyruvate is a monocarboxylate critically located at the crossroads of glycolytic and fermentative metabolism. This monocarboxylate is a three-carbon metabolite produced in the cytosol of living cells by the breakdown of glucose, a six-carbon hexose through the glycolytic pathway. Through the lactate dehydrogenase (LDH) catalysis, it is reduced to lactate, a three-carbon metabolite. Depending on its concentration gradient across the plasma membrane, it can be exported to the extracellular space mediated by the proton-coupled monocarboxylate transporters (MCTs). Furthermore, driven by the mitochondrial pyruvate carrier (MPC), it feeds the mitochondrial tricarboxylic acid cycle (TCA), generating ATP or serving as a building block for cell growth. Therefore, pyruvate is a core metabolite at the intersection of glycolysis and oxidative phosphorylation (OXPHOS), displaying catabolic and anabolic functions. In oxidative cells, such as neurons, halting the provision of pyruvate into mitochondria leads to a rewiring of oxidative metabolism^3^ and changes in synaptic transmission and neuronal excitability^4^^,^^5^ in both *in vitro* and *in vivo* models. Interestingly, pyruvate supplementation has been used in models of kindling and Alzheimer’s as a metabolic corrector.^6^^–^^8^

Commonly, the fate of pyruvate metabolism has been traced using mass spectrometry, nuclear magnetic resonance (NMR), isotopic labelling, and colorimetric techniques. Although these approaches have proven useful, they present several limitations: low spatial resolution; requiring thousands of cells per data point and low temporal resolution; making it challenging to capture rapid events in the order of seconds and consume the sample; precluding before-and-after experimental designs and requiring expensive equipment; and preventing broad accessibility to technology. Accordingly, fluorescent protein-based reporters have been engineered to measure pyruvate metabolism in single cells with second-scale temporal resolution, providing an easily detectable readout, with the potential to be well-calibrated without consuming the sample and the use of permeabilization procedures.

**Fig. 1:**
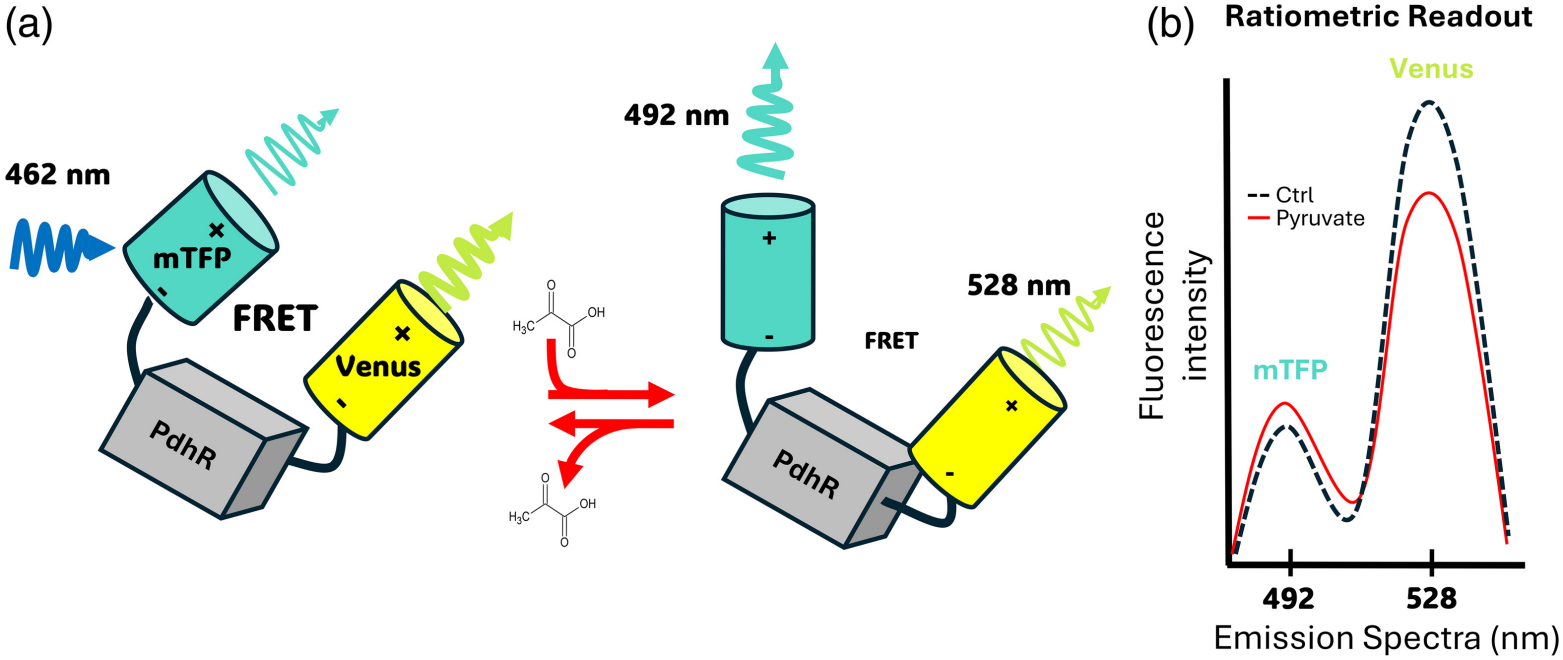
Schematic representation of the Pyronic and its spectroscopic properties. (a) Pyronic consists of the metabolite-binding transcriptional regulator PdhR flanked by two fluorescent proteins, mTFP (donor), and Venus (acceptor). In the absence of ligand (left), the close proximity of the fluorophores enables efficient Förster resonance energy transfer (FRET): excitation at 462 nm predominantly excites mTFP, leading to strong Venus emission at 528 nm. Binding of pyruvate to PdhR (center) triggers a conformational change that increases the donor–acceptor distance and/or alters their relative orientation, thereby lowering FRET efficiency. This rearrangement increases mTFP emission at 492 nm and decreases Venus emission. (b) Pyronic yields a ratiometric readout. The dashed black trace represents the basal (ligand-free) condition, whereas the red trace shows the fluorescence spectrum after pyruvate addition, demonstrating reciprocal changes in fluorescence intensity at 492 nm (mTFP) and 528 nm (Venus).

Genetically encoded fluorescent indicators are fusion proteins engineered from a binding-ligand domain and a fluorescent reporter module.^9^ The first and most common architecture is the Förster Resonance Energy Transfer (FRET).^10^ In these types of indicators, the binding of the metabolite of interest induces a conformational change that alters the distance and orientation between the donor and acceptor fluorescent proteins, thereby affecting the FRET efficiency [Figs. 1(a) and 1(b)].

These changes in steady-state fluorescence intensity can be easily monitored using fluorescence microscopy, providing valuable readouts of relative levels, concentration, and fluxes. These fluorescent indicators have emerged as a convenient tool to assess rapid intracellular metabolite dynamics, and their readout is amenable to calibration for quantitative assessment of intracellular metabolites.^9^ However, due to the complexity of cellular metabolism in terms of metabolite transport and production/consumption, precisely controlling their intracellular concentration in a noninvasive manner is challenging.

**Fig. 2:**
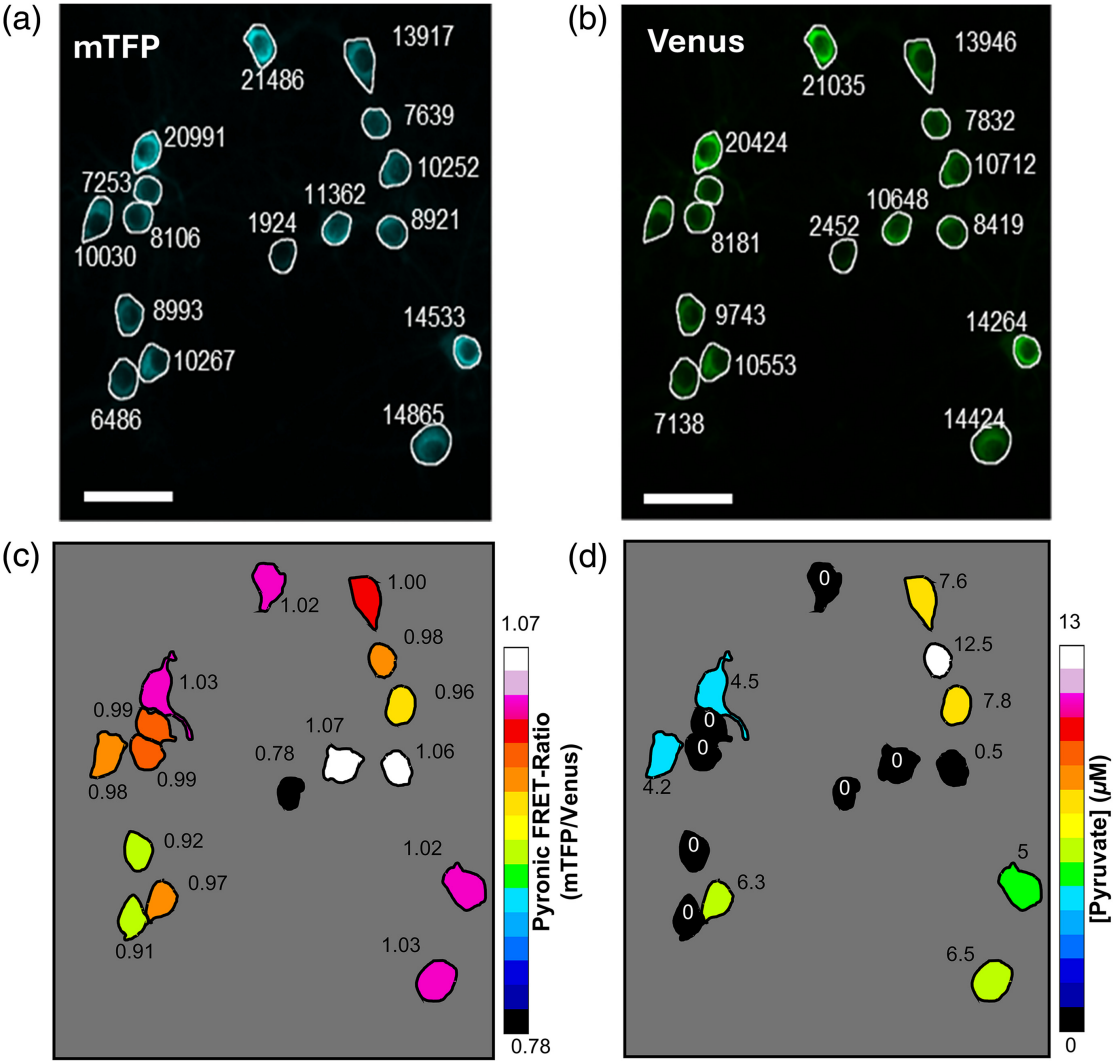
FRET ratio does not directly reflect intracellular pyruvate concentration. (a) and (b) Confocal micrograph showing the cytosolic distribution of Pyronic in cultured neurons. Arbitrary unit of fluorescence intensity of (a) mTFP and (b) Venus channels of Pyronic. (c) Pseudocolour representation of Pyronic FRET ratio. (d) Pseudocolour map of the intracellular pyruvate concentration at single-cell resolution obtained using the two-point calibration protocol. The procedures involved in obtaining an absolute number will be disclosed below. All the images were acquired under conditions of 2 mM glucose and 1 mM lactate. Scale bar 30  μm.

The most popular readout from fluorescent indicators is fluorescence intensity, which is not inherently quantitative. However, using the same baseline signal, it is possible to make quantitative comparisons in the same cell in a before-and-after experimental design.^11^^–^^13^ Nonetheless, absolute numbers are needed because the behavior of the indicator is affected by the cellular microenvironment,^14^^–^^16^ protein folding and chromophore maturation,^17^^,^^18^ equipment optics,^19^ and other factors,^20^ making it impossible to compare cells and sample batches directly using fluorescence intensity or FRET-ratio (Fig. 2). Converting fluorescence data into metabolite concentrations permits wider comparisons between studies and experimental conditions, as well as quantitative analysis of metabolic networks. Therefore, quantitative assessment is highly desirable to avoid artefacts. If the intracellular level of a given metabolite can be manipulated—either depleted or loaded—a one-point calibration can be performed by measuring the apo- or saturated-indicator signal. In both cases, additional information, such as the dynamic range and $K_{D}$ from the original characterization using a purified fluorescent indicator, is required. If it is possible to obtain both the apo- and saturated-indicator signal by manipulating the metabolite’s concentration from the extracellular space, a two-point calibration can be performed, relying on experimental data except for the pre-established $K_{D}$. In addition, full fluorescent indicator calibration is another alternative that involves detergents to permeabilize the plasma membrane and the superfusion of intracellular-emulating solutions, containing known metabolite concentrations, offered in a stepwise sequence. However, these procedures were found to be impractical and cumbersome due to rapid cellular swelling and sensor loss.^21^^,^^22^ In this regard, among the available experimental strategies for obtaining a quantitative assessment of a given intracellular metabolite, two-point calibration offers a good balance between invasiveness and the level of precision required for accurate evaluation of intermediate metabolism.

With the aim of monitoring and quantifying pyruvate dynamics with single-cell resolution, we engineered Pyronic,^23^ a pyruvate-sensitive genetically encoded FRET-based sensor. This fluorescent indicator have been instrumental to study brain energy metabolism *in vivo* with cellular resolution,^24^^–^^26^ determine the affinity of monocarboxylate transporter,^12^ identification of a monocarboxylate transporter associated with type 2 diabetes,^27^ assessment of mitochondrial pyruvate consumption in neurons,^28^ energization endothelial cells motility,^29^ the influence of chronic exposure of ketones bodies in astrocytic mitochondrial metabolism,^30^ and the identification of neuronal signals of metabolic coupling.^31^^,^^32^

Here, we describe a minimally invasive protocol that allows for two-point calibration of Pyronic FRET signal from neurons. This protocol can be performed *in vitro* and *ex vivo* preparations and extended to other brain and nonbrain cells.

## 2 Pyronic, a FRET-based Fluorescent Indicator for Pyruvate

Pyronic is a genetically encoded FRET-based sensor for pyruvate, based on the *Escherichia coli* transcriptional regulator PdhR, flanked with the fluorescent proteins mTFP and Venus, as FRET donor and acceptor fluorophores, respectively [Fig. 1(a)]. Pyruvate binding triggers a conformational change that lowers FRET efficiency, thereby increasing donor (mTFP) fluorescence and concomitantly decreasing acceptor (Venus) emission detected in the FRET channel [Fig. 1(b)]. The Pyronic affinity constant ($K_{D}$) for pyruvate is 107±13  μM. Due to the dependency of the FRET ratio on pyruvate concentrations, Pyronic can detect pyruvate concentrations ranging from 10 to 1000  μM, which nicely covers the entire physiological range of cytosolic pyruvate in mammals and other systems, typically in the range of 20 to 200  μM.^33^ Pyronic is highly selective, showing no sensitivity for other physiologically relevant monocarboxylates, and it is insensitive to pH within the physiological range.^23^ Pyronic shows ∼20% of maximum dynamic range ($\Delta R_{\mathrm{MAX}}$) on purified protein and 40% in astrocytes, HEK293, COS7, and MDA-MB-231 cells.^12^^,^^23^^,^^27^^,^^33^ However, in neurons, Pyronic showed a differential dynamic range between 20% and 35%.^25^^,^^28^^,^^34^ The variation in dynamic range stems from the different expression levels reached with the selected gene-delivery methods. Transfection using cationic lipids typically introduces enough gene copies to yield high levels, allowing it to reach a dynamic range of roughly 40%, whereas viral vectors usually achieve only 20% to 35%. Therefore, a two-point calibration is ideal for calibrating the fluorescence signal, because the dynamic range depends on the indicator expression level attained in each individual cell. Determining the dynamic range on a cell-to-cell basis prevents over- or underestimation of the fluorescence window used to accurately calculate intracellular pyruvate concentrations.

## 3 Rationale for the Two-point Noninvasive Calibration of Pyronic

Cytosolic steady-state level of pyruvate is sustained by glycolytic production, the equilibrium through the LDH reaction with lactate, and the transport across the plasma and the inner mitochondrial membranes by the MCT2 and MPC transporters, respectively. We have devised a simple two-point calibration protocol based on this simplified metabolic arrangement to assess the minimum and maximum Pyronic signal.

Given the high-affinity MCT2 transporter and the pyruvate oxidation within mitochondria, cytosolic pyruvate quickly drops, and Pyronic fully desaturates when neurons are superfused with a saline solution without energy sources, allowing the $R_{\mathrm{MIN}}$ (mTFP/Venus minimal ratio, apo-Pyronic state) to be obtained. On the contrary, when MCT and MPC are pharmacologically blocked, whereas glycolysis is boosted to increase pyruvate production, cytosolic pyruvate concentration saturates Pyronic to obtain the $R_{\mathrm{MAX}}$ (mTFP/Venus maximal ratio, fully saturated-Pyronic state). To afford this, we bath neurons with a saline solution containing 10 mM pyruvate, 1 mM lactate, 2 mM glucose, 1  μM AR-C155858 or AZD3965, both MCT1/2 blockers,^35^^,^^36^ 10  μM UK-5099, an MPC blocker,^37^ and 5 mM sodium azide. Sodium azide is a mitochondrial complex IV inhibitor that stops the electron transport chain (ETC), halting mitochondrial ATP production. However, due to the drop in cytosolic ATP, glycolysis is boosted as a metabolic compensation,^38^ favoring pyruvate production and therefore its intracellular accumulation. All together, these procedures performed at the end of each experimental run revealed the dynamic range of Pyronic $\Delta R_{\mathrm{MAX}}$ ($R_{\mathrm{MAX}}-R_{\mathrm{MIN}}$), uncovering its full dynamic range in each cell from every batch of samples. Determining the full dynamic range of Pyronic from each cell is important, especially when a heterogeneous fluorescence signal is detected, depending on the gene-delivery system. Although this design was developed for primary hippocampal neurons, it could be applied to any other cell type by simply adjusting the MCT inhibitor to target the appropriate isoform.

## 4 Primary Hippocampal Neurons from Mice

Primary cultures offer a favorable balance between experimental control and physiological relevance. Consequently, this model allows direct and controlled access to the cellular environment, enabling the establishment of causality for a given pharmacological, nutritional, optogenetic, or genetic perturbation. This makes it an ideal model to elucidate the nuances of the mechanisms underlying a phenomenon of interest. In addition, we have gravitated to co-culture embryonic hippocampal neurons and astrocytes to promote *in vitro* differentiation and metabolic cooperation.^28^^,^^39^^,^^40^ This type of co-culture of neurons and astrocytes has been used to unveil a plethora of neuronal signals related to metabolic coupling, which have been validated *in vivo*.^25^^,^^41^

A detailed description of the steps involved in sample preparation for the imaging experiment will be provided below.

### 4.1 Experimental Model and Subject Details

Hybrid females F1 C57BL/6J × CBA/J mice aged 2 to 6 months carrying 6 to 9 embryos were used. All animals were housed in standard pathogen-free (SPF) conditions with a 12:12-h light/dark cycle at room temperature (20°C±2°C) and had free access to food and water. All experimental procedures were approved by the Centro de Estudios Científicos Animal Care and Use Committee following the recommendations of the Guide for the Care and Use of Laboratory Animals, Institute of Laboratory Animal Resources, National Research Council.

## 5 Drugs, Reagents and Equipment

### 5.1 Euthanasia

• Isoflurane (100%, UPS grade, Baxter cat. #10019036060).
• Anesthesia chamber.
• Cotton pads or gauze.
• Pasteur pipette.
• Cervical dislocation support base.
• Dedicated protective clothing for the dissection area (e.g., face mask, gloves, lab coat).
• Disposal container for carcasses.

### 5.2 Dissection

• Stereo microscope (e.g., Olympus SZ61).
• Large, sharp scissors.
• Dissection pins.
• Rat-tooth forceps (toothed forceps).
• Curved forceps and fine-tip forceps.
• Fine scissors.
• Two Petri glass dishes (100 mm).
• Two 1-mL conical tubes, filled with cold Hanks’ Balanced Salt Solution (HBSS).
• Two 50-mL Falcon tubes, filled with cold HBSS.
• A Styrofoam box filled with ice.
• Laminar flow cabinet.

### 5.3 Neuronal Culture

• 30 mm Petri dishes (or alternatively, 6-well plates).
• 15-mm glass coverslips.
• Poli-l-lysine (Sigma, cat. # P4832).
• Trypsin-EDTA (0.5%) in DPBS (10X) (Capricorn, Cat. TRY-1B10).
• Neurobasal-A medium, no d-glucose, no sodium pyruvate (Gibco, cat. # A2477501).
• d-Glucose solution (Gibco, cat. # A2494001).
• Sodium pyruvate (Gibco, cat. # 11360070).
• GlutaMAX Supplement (Gibco, cat. # 35050061).
• B-27 Supplement (Gibco, cat. # 17504044).
• Penicillin/streptomycin (Gibco, cat. # 15140122).

### 5.4 Neuronal Transduction with Pyronic

• Pyronic (Addgene: Plasmid #51308).
• AAV-DJ-hSyn-Pyronic.
• Hippocampal neuronal-astrocytic co-cultures.

**Note:** The plasmidial DNA encoding for Pyronic can be requested from Addgene (Addgene: Plasmid #51308). However, to deliver it in a cell-specific manner (i.e., targeting neurons under the hSyn promoter) or to clone it into an adeno-associated virus (AAV) particle, further cloning is required (Addgene: Plasmid #50465). AAV particle production was performed using the AAV-Dj Helper Free Packaging System from Cell Biolabs Inc. Both plasmidial DNA and AAV are typically classified as risk factor 1 agents by the US National Institutes of Health (NIH); therefore, handling must comply with NIH guidelines.

### 5.5 Saline Solutions

• ${\mathrm{ddH}}_{2}O$.
• NaCl (Sigma Aldrich, CAS# 7647-14-5).
• KCl (Sigma Aldrich, CAS# 7447-40-7).
• ${\mathrm{CaCl}}_{2}$ (Merck, CAS# 10035-04-8).
• ${\mathrm{MgCl}}_{2}$ (Merck, CAS# 7791-18-6).
• HEPES (Merck, CAS# 7365-45-9).
• ${\mathrm{NaHCO}}_{3}$ (Merck, CAS# 144-55-8).
• NaOH (Merck, CAS# 1310-73-2) to titrate the saline solution to the corresponding pH.

**Caution**: NaOH is a strong base. We recommend using a 1-M stock solution. Handle with care and wear appropriate PPE, including gloves, a lab coat, and eye protection.

• pH meter (e.g., Extech Model 321990).
• Osmometer (e.g., Advanced instrument Model 3250).
• Plate stirrer.
• Analytical balance (laboratory scale).
• 1-L glass bottles.
• Beakers, volumetric flasks, spoons and spatulas, magnetic stir, plastic plates for measuring compounds, etc.

### 5.6 Drugs

• Sodium azide (Sigma Aldrich, CAS # 26628-22-8).
• UK-5099 (MedChemExpress, CAS # 56396-35-1).
• AR-C155858 (MedChemExpress, CAS # 496791-37-8).
• AZD 3965 (MedChemExpress, CAS # 1448671-31-5).
• Dimethyl sulfoxide (Sigma-Aldrich, CAS # D8418).

### 5.7 Imaging Setup

• Laser-scanning microscope (e.g., Fluoview FV1000).
• 440-nm solid-state laser (405 nm solid-state laser can be used instead; however, only with 50% efficiency in excitation).
• 20× water-immersion objective (N.A. 1.0).
• Excitation-emission cube: 405 to 440/515 dichroic mirror.
• Emission split: SDM510 dichroic mirror.
• Band-pass filters; mTFP: 465 to 495 nm, Venus: 535 to 565 nm.

### 5.8 Recording Chamber and Perfusion System

• Recording chamber (Warner instruments, RC-26GLP cat. #64-0236).
• Bipolar Temperature Controller Model CL-100 (Warner Instrument, cat. #W464-0352).
• Liquid Cooling System Model LCS-1 (Warner Instrument, cat. #W464-1922).
• Dual In-line Solution Heater/Cooler Model SC-20 (Warner Instrument, cat. #W464-0353).
• Thermistor Model TA-29 (Warner Instrument, cat. # W464-0107).
• Peristaltic and/or vacuum pump (Fisherbrand™ Variable-Flow Peristaltic Pumps). Alternatively, gravity-driven inflow can be used.
• 5% ${\mathrm{CO}}_{2}/95\%$ air gas mixture.

### 5.9 Software

• Image acquisition and analysis software (dedicated software provided by the microscope or μManager ImageJ).

## 6 Equipment Setup

### 6.1 Imaging Setup

The imaging setup must include all the previously detailed optics to effectively record Pyronic’s signal. Alternative setup arrangements, such as those based on LED illumination, optoscan monochromators, and optosplits, are also suitable and amenable to FRET recordings. A quick check out of all the electronic systems, such as shutters and camera idling, as well as excitation and collection of the emitted photons, is highly recommended in an early introductory session.

Although this protocol is based on an upright microscope, similar results are obtained when using an inverted system. With inverted systems, no major considerations are needed, except for using the correct immersion oil or water as required by the objective specifications.

On the day of recording, it is recommended to turn on all the equipment, including the microscope, light source, temperature regulator, and computer, in advance. Typically, it takes about 30 min for the microscope’s body temperature and light source power to stabilize. To minimize focus drift during experiments, an autofocus system can be attached to the microscope. The room should be darkened, and the temperature should be maintained at a stable level; using an air conditioning system is advisable.

### 6.2 Recording Chamber

Disassemble the recording chamber and add vacuum grease to the chamber slit to secure a good seal. Inflow and outflow perfusion lines must be connected to the corresponding peristaltic or vacuum pumps and tested for proper functioning. The solution overflow regularly occurs because of poor arrangement of the tubing in the pump slits or connections.

### 6.3 Perfusion System

**Fig. 3:**
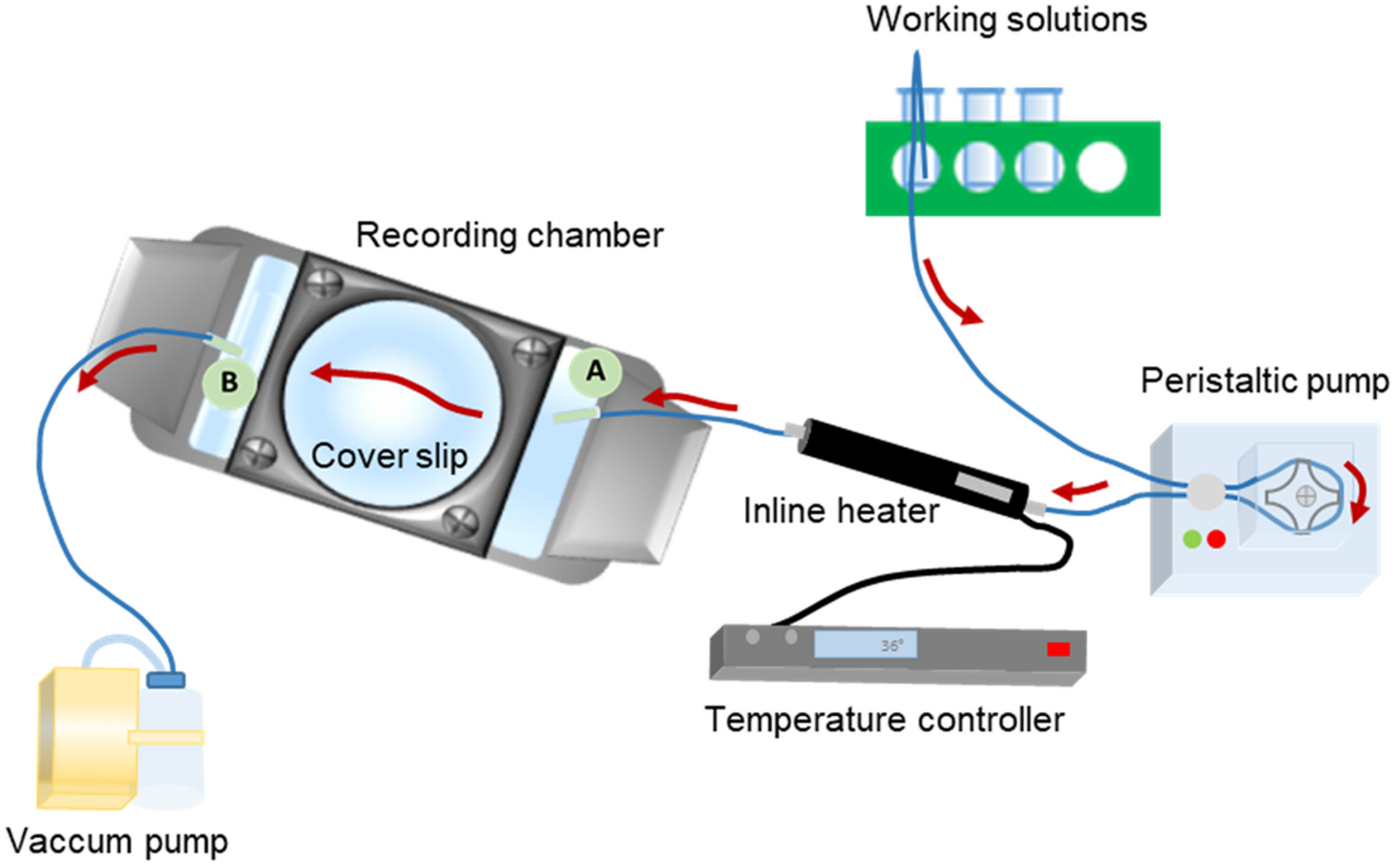
Open perfusion setup for single-cell recordings. Working solution is drawn from a reservoir through 1.6-mm inner-diameter silicone tubing and driven by a peristaltic pump toward the recording chamber inlet. When temperature control is required, the line passes through an inline heater placed as close as possible to the chamber. The solution bathes the fully accessible recording chamber, allowing localized drug application with a pipette when continuous flow is unnecessary. Effluent exits the chamber via an outflow line to a waste container or vacuum trap. Red arrows indicate the direction of the buffer flow.

We use an open perfusion system that collects the working solution at one end, bathes the recording chamber, and then discards the solution at the other end (Fig. 3). Another feature of our perfusion system is that the recording chamber is fully accessible with a pipette, which is beneficial for incubating drugs directly within the chamber when continuous perfusion is unnecessary or should be avoided. This situation may arise when testing a small amount of a drug or during extended preincubation times. However, it is important to note that stopping the perfusion system when using a bicarbonate-buffered solution can affect the pH. Therefore, we recommend using a HEPES-buffered solution in these cases. Alternatively, recirculation systems can be employed with bicarbonate-buffered solutions when dealing with small drug quantities, or prolonged incubation times cannot be avoided. In this setting, both ends of the perfusion system must be placed in the same cylinder tube.

Typically, the perfusion is established using 1.6-mm diameter silicone tubing. The first segment (hereafter, the inflow) goes from the cylinder tube containing the working solution, passes through the peristaltic pump, and ends at the inlet of the recording chamber. If the temperature needs to be controlled, an inline heater should be placed between the peristaltic pump and the recording chamber. In this case, the inflow tubing is connected to the inline heater after being wrapped through the peristaltic pump slots. We recommend strategically placing the inline heater close to the recording chamber. Finally, a short piece of silicon tubing is needed to connect the inline heater to the inlet port of the recording chamber. The second segment, hereafter referred to as the outflow, extends from the outlet port of the chamber to the vacuum pump port or the disposal container.

### 6.4 Solutions

We commonly use bicarbonate buffer of the following composition (in mM): 112 NaCl, 3 KCl, 1.25 ${\mathrm{CaCl}}_{2}$, 1.25 ${\mathrm{MgSO}}_{4}$, 10 HEPES, 24 ${\mathrm{NaHCO}}_{3}$, pH 7.2 adjusted with 1 M NaOH, and bubbled with 95% air/5% ${\mathrm{CO}}_{2}$. Alternatively, a bicarbonate-free solution of the following composition can also be used (in mM): 136 NaCl, 3 KCl, 1.25 ${\mathrm{CaCl}}_{2}$, 1.25 ${\mathrm{MgCl}}_{2}$, 10 HEPES, and pH 7.4 adjusted with 1M NaOH. We typically prepare 2 L of saline solution, which, depending on the experimental flow rate of the perfusion system, lasts ∼15  h of recordings. Importantly, we do not recommend using saline solutions older than 2 weeks, as the pH slowly changes, accompanied by the concomitant formation of a precipitate.

On the experimental day, fill a 100-mL volumetric flask with the saline solution of choice (bicarbonate- or bicarbonate-free-buffered) and supplement it with 2 mM glucose and 1 mM lactate. Next, split it into two 50-mL conical tubes, and add 10 mM pyruvate, 2  μM AR-C155858 or AZD 3965, 10  μM UK-5099, and 5-mM sodium azide into one of the two tubes. Finally, fill a third 50-mL conical tube with the saline solution of choice, but do not supplement with energy substrates. Once ready, all solutions must be placed in a rack by the imaging setup. Bicarbonate-buffered solutions must be bubbled with 5% ${\mathrm{CO}}_{2}/95\%$ air for at least 30 min to stabilize pH.

**Tip:** The experimental solution can be prepared after turning on the equipment to use the time more efficiently.

**Tip:** We use prepared 1-M stock solutions stored at −20°C for up to 6 months for glucose, lactate, and pyruvate. Drugs are prepared as 1000× stocks and kept at −20°C.

## 7 Procedure

### 7.1 Mixed Neuron-astrocyte Primary Cultures. (14 to 16 Days Before Experimental Day)

1. Pregnant mice at embryonic day 17.5 (E17.5) were euthanized by cervical dislocation following deep anesthesia with isoflurane, in accordance with institutional ethical guidelines and the ARRIVE principles. Under sterile conditions, 6 to 9 embryos were harvested and transferred to ice-cold HBSS supplemented with 5 mM glucose. Brains were rapidly isolated, and hippocampi were dissected under a stereomicroscope, ensuring complete removal of the meninges. Tissues were enzymatically dissociated in 1% trypsin-EDTA (Sigma-Aldrich) in HBSS for 15 min at 37°C. The enzymatic reaction was quenched by adding Neurobasal medium (Gibco) supplemented with 10 mM glucose, 2% B-27 supplement, 1% GlutaMAX, and 5% fetal bovine serum (FBS). Hippocampal cells were mechanically triturated and seeded onto 25-mm poly-d-lysine-coated glass coverslips at a density of 160,000  cells/mL. Cells were allowed to adhere for 2 h at 37°C in a humidified incubator (95% air, 5% ${\mathrm{CO}}_{2}$). After this period, the plating medium was replaced with serum-free Neurobasal medium supplemented with 10 mM glucose, 2% B-27 supplement, 1% GlutaMAX, 2.5 and 10  μg/mL penicillin/streptomycin. Neuron-astrocyte co-cultures were maintained at 37°C in a humidified incubator (95% air, 5% ${\mathrm{CO}}_{2}$), with two-thirds of the medium exchanged every 3 days.

### 7.2 Pyronic Transduction in Primary Hippocampal Neurons (5 to 7 Days Before Experimental Day)

2. Add the AAV-DJ-hSyn-Pyronic particles directly to the culture medium 5 to 7 days before the experimental session and mix thoroughly. This procedure results in more than 80% of neurons expressing the sensor. Alternatively, lentiviral vectors or conventional lipofection procedures have been successfully used.^23^^,^^28^

**Tip:** The number of viral particles will depend on the titer of the viral preparation. We recommend using the lowest amount of virus that allows for good expression and a reasonable signal-to-noise ratio, without any observable inclusion bodies or signs of saturation.

**Tip:** AAV serotype 2 is commonly used in cultured brain slices and stereotactic procedures due to its broad tissue tropism, enabling the efficient transduction of neurons. However, its transduction efficiency in primary neuronal cultures is limited. To overcome this, we cloned Pyronic into an AAV vector expressing the AAV-DJ capsid, a synthetic hybrid serotype generated by DNA family shuffling of eight wild-type AAVs (AAV1–9). AAV-DJ combines the advantageous properties of these parental serotypes to achieve high transduction efficiency across a broad range of cell types, including neurons. It exhibits superior *in vitro* infectivity compared to natural serotypes, making it an ideal vector for delivering genes to neurons in both dissociated cultures and tissue slices.^42^

**Note:** In our hands, lipid transfection (Lipofectamine) is also possible, but the efficiency is low (<10%).

**Tip:** The gene-delivery system determines the number of copies of the DNA delivered into the cell and, consequently, is one factor that determines the levels of expressed protein and the signal-to-noise ratio. Lipofection displays low transfection efficiency but allows obtaining heterogeneous and high fluorescence signals in some cells, resulting in $\Delta R_{\mathrm{MAX}}$ values closer to the 40% dynamic range previously reported in various cellular systems. On the other hand, lentiviral particles offer high gene-delivery efficiency but yield a lower signal-to-noise ratio due to the limited gene copies that can be inserted into the genome, with a $\Delta R_{\mathrm{MAX}}$ of ∼20%. In our experience, adeno-associated viruses performed optimally, effectively balancing gene-delivery efficiency and signal-to-noise ratio, achieving a $\Delta R_{\mathrm{MAX}}$ of around 35%.

**Tip:** Cytosolic pyruvate concentrations have been reported in the micromolar range.^28^^,^^33^ Therefore, performing experiments in cells that display fluorescent indicators expressed in the same range could produce buffering effects.^43^ This is the case for plasmids with strong promoters such as CMV or CAG that produce a micromolar range of fluorescent indicators.^44^^,^^45^ Under these conditions, Pyronic could act as a chelator, decreasing the baseline levels of pyruvate and potentially affecting cell physiology. In addition, cells overexpressing fluorescent indicators also exhibited significant impairment in mitochondrial oxidative respiration and changes in proteomics profile, suggesting broader mitochondrial dysfunction.^46^ To minimize these scenarios, the use of gene-delivery systems such as adeno-associated virus with expression driven by cell–type-specific promoter will minimize these unwanted effects.

### 7.3 Imaging Pyronic (On the Experimental Day)

3. Take the 25-mm glass coverslip from the petri dish and mount it in the recording chamber. Once the chamber is closed, add 0.5 mL of saline solution supplemented with 2 mM glucose and 1 mM lactate to prevent the cells from drying out.
4. Place the recording chamber in the microscope stage and connect the inflow and outflow lines. Inline heaters are typically positioned before the recording chamber if temperature control is desired. A thermistor is also recommended in the recording chamber to monitor temperature variations throughout the experiment.

**Tip:** Experiments performed at 35°C to 36°C are often noisier and more technically challenging. As temperature increases, the metabolic rate generally accelerates due to enhanced enzymatic activity, membrane fluidity, and faster molecular diffusion.^47^ We recommend running a few experiments at room temperature when inexperienced with live imaging. This favors more stable recordings and improves the degree of confidence in handling the technique. To avoid degassing, heat the solutions in a water bath.

5. Turn on the perfusion system. The volume of superfusate in the chamber is ∼0.5  mL. Keep a stable and continuous perfusion rate of about 2  mL/min, which secures an approximate turnover time of the fluid in the chamber of about 15 s. Quick wash-in of sugars and drugs is desired for kinetic analysis of pyruvate dynamics; therefore, turnover times must be maintained high.

**Tip:** Cells can also be superfused by gravity. Height has to be arranged accordingly to ensure a constant flux in the chamber, which must be correctly equalized to the efflux rate. In addition, the speed of gravity-based perfusion decreases when saline solutions are used. The variation in the speed may induce substantial changes in temperature when an in-line heater is positioned before the recording chamber.

**Tip:** When culturing primary brain cells, we typically use a glucose concentration of 10 mM, but experiments are conducted in 2 mM glucose. Therefore, it is recommended to allow the cells to equilibrate to the new conditions in about ten minutes. Notably, we observed that when fluorescence recordings of glucose began immediately after mounting the coverslip in the recording chamber, there was a gradual transition in intracellular glucose concentration toward a new steady state. This transition occurs as the cells need to reestablish glucose gradients. This issue may be even more pronounced when neurons are cultured in standard 25-mM glucose medium but are recorded at lower glucose concentrations.

6. Find the focal plane under bright field illumination. Using a 20× objective is sufficient to record from neuronal bodies in primary culture. This also secures about 20 cells per field of view, improving data collection per experiment. The 40× objective is equally suited, but fewer cells are recorded per imaging session.
7. Switch on a light source, excite the sample at 440 nm, and scan the plate using either of the two emission channels (mTFP or Venus). Alternatively, the sample can be scanned first with 480-nm excitation light and the Venus filter. Under the fluorescence microscope, Pyronic expression should be observed as homogeneously distributed in the cytosol and with explicit nuclear exclusion [Figs. 2(a) and 2(b)]. Bright spots indicate protein aggregation presumably due to overexpression and should be avoided.

**Tip:** When Pyronic is allowed to express for too many days (in our experience, more than 12 to 14 days), the sensor coalesces into large pyruvate-insensitive aggregates. This negatively impacts the sensor’s dynamic range and decreases the signal-to-noise ratio. We do not recommend imaging these neurons.

8. Take a snap with the imaging software and check for the level of fluorescence in each channel. Modify the illumination intensity or detector sensitivity accordingly to improve photon collection for both emission channels, preventing saturated pixels. As a general rule, illumination should be kept to the minimum amount of light that generates a reasonable signal-to-noise ratio. Excessive exposure can lead to photobleaching and/or phototoxicity, as well as contamination by autofluorescence, so it should be prevented.

**Tip:** The two fluorescent proteins used in Pyronic are resistant to photobleaching; however, low illumination exposure times and power are always advisable. Under optimal excitation and photon collection conditions, we have performed experiments for up to 1 h without observing any signs of quenching in the fluorophores, cellular photodamage, or significant drift. Nonetheless, when illumination has to be strong, lower the acquisition frequency to minimize bleaching. Most commonly, intense illumination is needed when the Pyronic expression level is low; therefore, the transduction process may need to be reassessed.

**Tip:** The excitation intensity, exposure time, and detection gain must remain consistent throughout the experiment. Although adjusting excitation intensity or exposure time during recording might be corrected by the Pyronic ratio, altering the detection gain affects each emission channel nonlinearly. These changes ultimately influence the Pyronic ratio and the results of the experiments.

9. Adjust imaging frequency according to the expected kinetics of the question at hand. Most commonly, frames every 5 to 10 s are sufficiently fast to follow the metabolic response. Equipped with a 20× objective, images are taken regularly at 512×512  pixel resolution. However, 320×320  pixel resolution also works well. Larger images do not enhance the Pyronic data; they only capture unnecessary details. The image depth is 16-bit, providing a wide range of tones per pixel. Nonetheless, 8-bit images are equally well suited.

**Tip:** Depending on the signal-to-noise ratio, binning might be advisable.

**Tip:** Most dedicated imaging software permits monitoring the experiment by a live plot of the Pyronic ratio (mTFP/Venus). This allows for real-time assessment of pyruvate dynamics, which eases the recognition of steady states or the effect of incubation with different saline solutions.

10. Start the imaging and wait for approximately 5 to 10 min, or until a stable baseline is reached. Ensure that the perfusion system runs steadily and that the solution volume trapped in the chamber and the temperature are constant during the experiment.

### 7.4 Pyronic Two-point Calibration

11. From a suitable field of view, with neurons expressing Pyronic but not exhibiting saturated pixels [Figs. 2(a) and 2(b)], obtain a stable baseline and then switch to the solution without extracellular carbon sources for about 5 to 10 min to reach the minimum Pyronic signal ($R_{\mathrm{MIN}}$ – apo-Pyronic state).

**Tip:** The time may vary from preparation to preparation due to the degree of spontaneous activity in the cultures. This step may take even longer if the experiments are performed in *a priori* silenced neurons (e.g., TTX treatment).

**Fig. 4:**
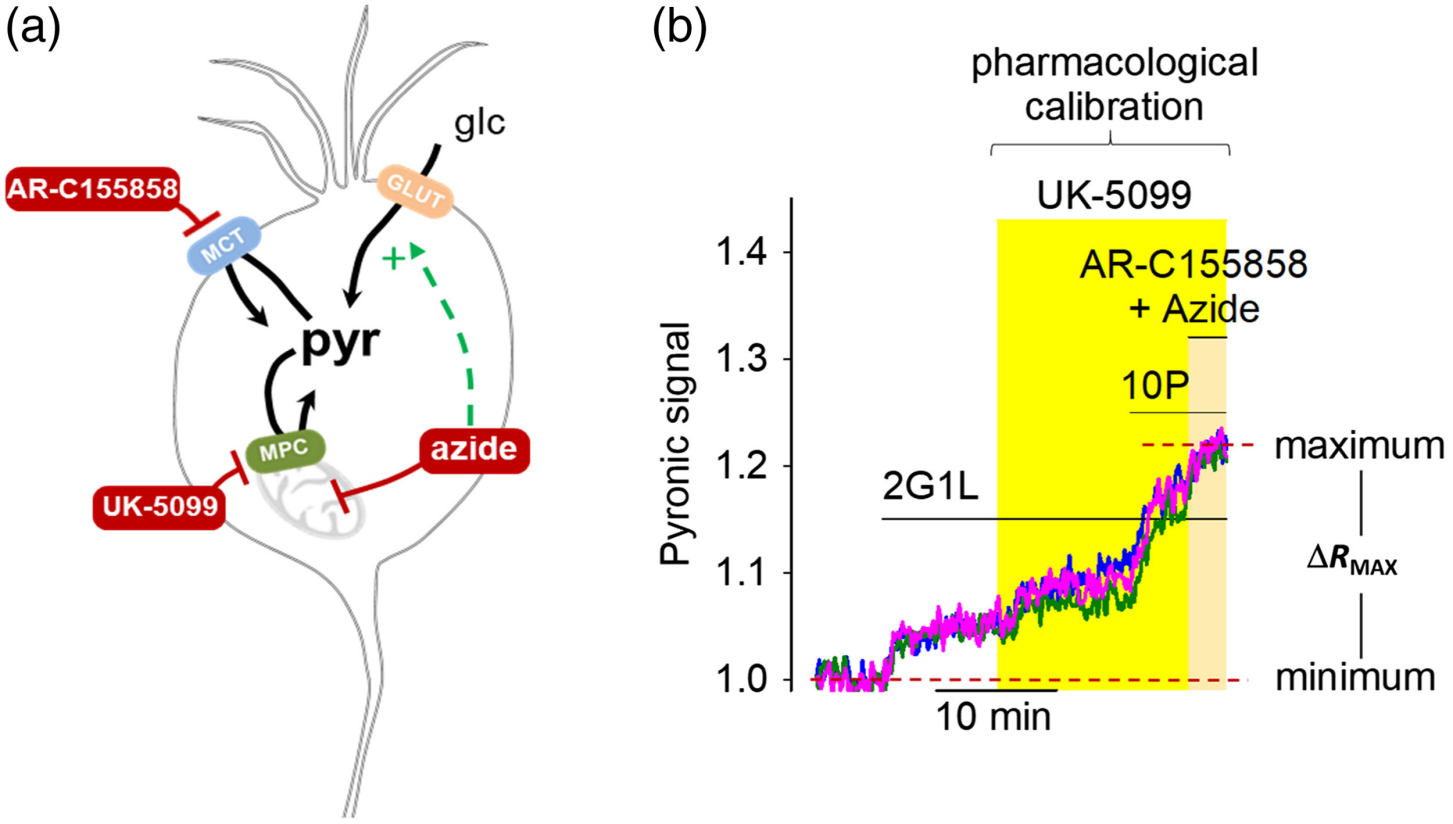
Pyronic two-calibration protocol. (a) Schematic representation of the pharmacological protocol used to accumulate intracellular pyruvate. Glucose (glc) and lactate enter the neuron via GLUT and MCT transporters, respectively. Pyruvate is transported into mitochondria by the MPC. Pharmacological agents used include 1  μM AR-C155858 (MCT inhibitor), 10  μM UK-5099 (MPC inhibitor), and 5 mM azide (OXPHOS inhibitor). (b) Representative traces of Pyronic signal from three single neurons during the calibration protocol. The $R_{\mathrm{MIN}}$ (minimum) is established by removing extracellular glucose and lactate (2G1L condition). The $R_{\mathrm{MAX}}$ (maximum) is then achieved by sequential application of 10  μM UK-5099, 10 mM pyruvate (10P), 1  μM AR-C155858, and 5 mM Azide in the presence of 2 mM glucose and 1 mM lactate. The dynamic range is defined by $\Delta R_{\mathrm{MAX}}=R_{\mathrm{MAX}}-R_{\mathrm{MIN}}$.

**Note:** Pyronic does not exhibit proton sensitivity, which is important because certain protocol steps—such as 5 mM azide—can produce acute intracellular pH changes. Therefore, the presented protocol is not advisable to be performed using pH-sensitive fluorescent indicators for pyruvate.^33^^,^^48^^–^^50^

12. Next, switch to a saline solution containing 5-mM sodium azide, 1  μM AR-C155858 or AZD 3965, and 10  μM UK-5099, in the presence of 2 mM glucose, 1 mM lactate, and 10 mM pyruvate, for 5 min to reach the maximum Pyronic signal ($R_{\mathrm{MAX}}$ – saturated-Pyronic state) [Fig. 4(a)]. As one example, when intracellular pyruvate precursors and inhibitors are offered sequentially, the increment of Pyronic signal, from minimum to maximum, can be unveiled as shown in Fig. 4(b). The same maximum signal is obtained when the pharmacological calibration cocktail is administered together. Thus, we recommend this simplified single-step procedure when assessing the maximum Pyronic signal.
13. **Important note:** Assessing the maximum Pyronic signal is a final procedure, as cells must be incubated with a cocktail of drugs that bind with a high degree of affinity to their targets and presumably irreversibly modify metabolism. We strongly emphasize that the maximum point calibration procedure must be performed at the end of the experiment. Interpretation of the data afterward will be dubious otherwise.

14. Finalize the experiment and save the images for later analysis. If a live plot is unavailable, we recommend an on-the-fly analysis of the experiment before pursuing another one. Most dedicated imaging softwares are implemented with an analysis section where plots of the single channels can be evaluated. This quick visualization of the experiment allows for checking the correct responsiveness of the sensor and considering changes to the protocol, such as timing or illumination settings.

**Important note:** We strongly recommend performing the two-point calibration as a sequential two-step protocol at the end of the experiment. Nevertheless, depending on the question in mind, an *ad hoc* protocol must be designed. As stated in Sec. 3, this calibration procedure relies on the cellular response. Therefore, both the minimum and maximum Pyronic signals must be assessed when the conditions are most favorable, but without interfering with a clear-cut assessment of the biological question at hand. For instance, if the effect of a specific blocker of mitochondrial respiration is to be assessed, the minimal Pyronic signal must be evaluated before inhibiting mitochondrial respiration.

### 7.5 Data Analysis: Regions of Interest and Fluorescence Intensities Analysis

15. Open the file of images in an imaging analysis software or FIJI.^51^ Depending on the software setting, the image stack will be automatically split into two channels. If this is not the case, separate them accordingly.
16. Scroll back and forth through the image stack and look for changes in the X−Y axes, cellular volume and shape, and background fluorescence. Sometimes, small pieces of cultured tissue become detached from the glass coverslip and eventually cross the field of view.

**Tip:** If there is noticeable drift along the X−Y axes, registration plug-ins available for FIJI (i.e., template matching and slide alignment by Qingzong Tseng) can be used to re-align the image stack. However, based on our experience, only minor X−Y drift is typically observed after 1-h experiments.

**Tip:** Z-axis drift is more challenging to correct. It is essential to maintain a stable temperature for both the saline solutions and the room, as temperature fluctuations can significantly affect Z-axis drift. We recommend pre-heating experimental solutions to the desired temperature. Additional measures include using objective, chamber, or in-line heaters. If available, we also suggest incorporating autofocus systems. Furthermore, an air conditioning system can help stabilize the room temperature.

**Fig. 5:**
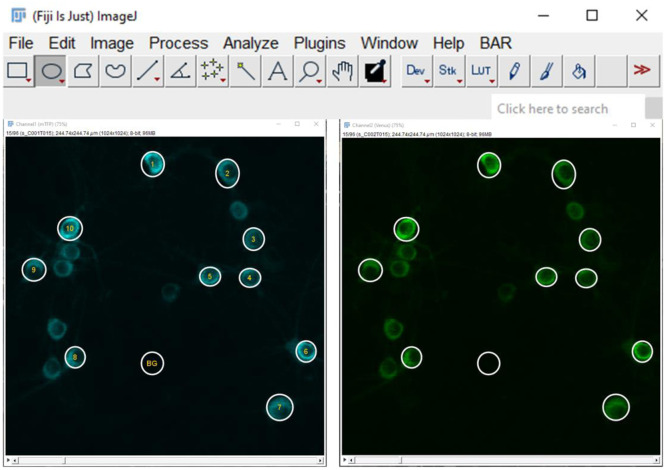
Screenshot of data acquisition for Pyronic FRET imaging using Fiji ImageJ. Cytosolic Pyronic mTFP and Venus signal from neurons. Regions of interest (ROIs) for analysis of specific individual cells (numbered circles) and the background region between cells (BG circle) were selected. The background area is used to subtract background fluorescence from each signal.

**Note:** Background fluorescence refers to the undesired light that reaches the detectors during imaging. This phenomenon can vary between different images and experiments due to several factors, including variations in acquisition settings, day-to-day instrument differences, sample-dependent autofluorescence (i.e., density of the astrocytic bed in neuron-astrocytes co-cultures, cleanliness of the glass coverslip), and fluctuations in light intensity (either from excitation light or the environment). To remove this non-specific light contribution, it is essential to subtract the background fluorescence from fluorescence measurements for each image.

**Note:** Pyronic displays a homogenous cytosolic pattern with a clear nuclear exclusion. The ratio of nucleus:cytoplasm area in neurons is high. Therefore, we prefer to cover the whole cell with the ROI in order not to lose any detectable signal from the cytoplasm.

17. Select regions of interest (ROI), including one ROI in an area without cells (Fig. 5). Commonly, we draw ROIs that are slightly larger than the cells to keep them within the ROI when a slight movement is presented. Scroll back and forth through the image stack, checking that cells remain within the ROI throughout the experiment.

18. Compute the average pixel intensity for each ROI on both channels. We do not recommend computing the maximum value or the sum of pixel intensities for each region, because it weights outlier pixels.
19. Copy and paste the fluorescence intensity values into an Excel sheet for further analysis. First, a background-corrected value is calculated by subtracting the background intensity from each channel in each ROI over time. Next, to obtain the normalized Pyronic signal, the complete experiment is divided by the average of the Pyronic signal at the minimum ratio ($R/R_{\mathrm{MIN}}$).

**Fig. 6:**
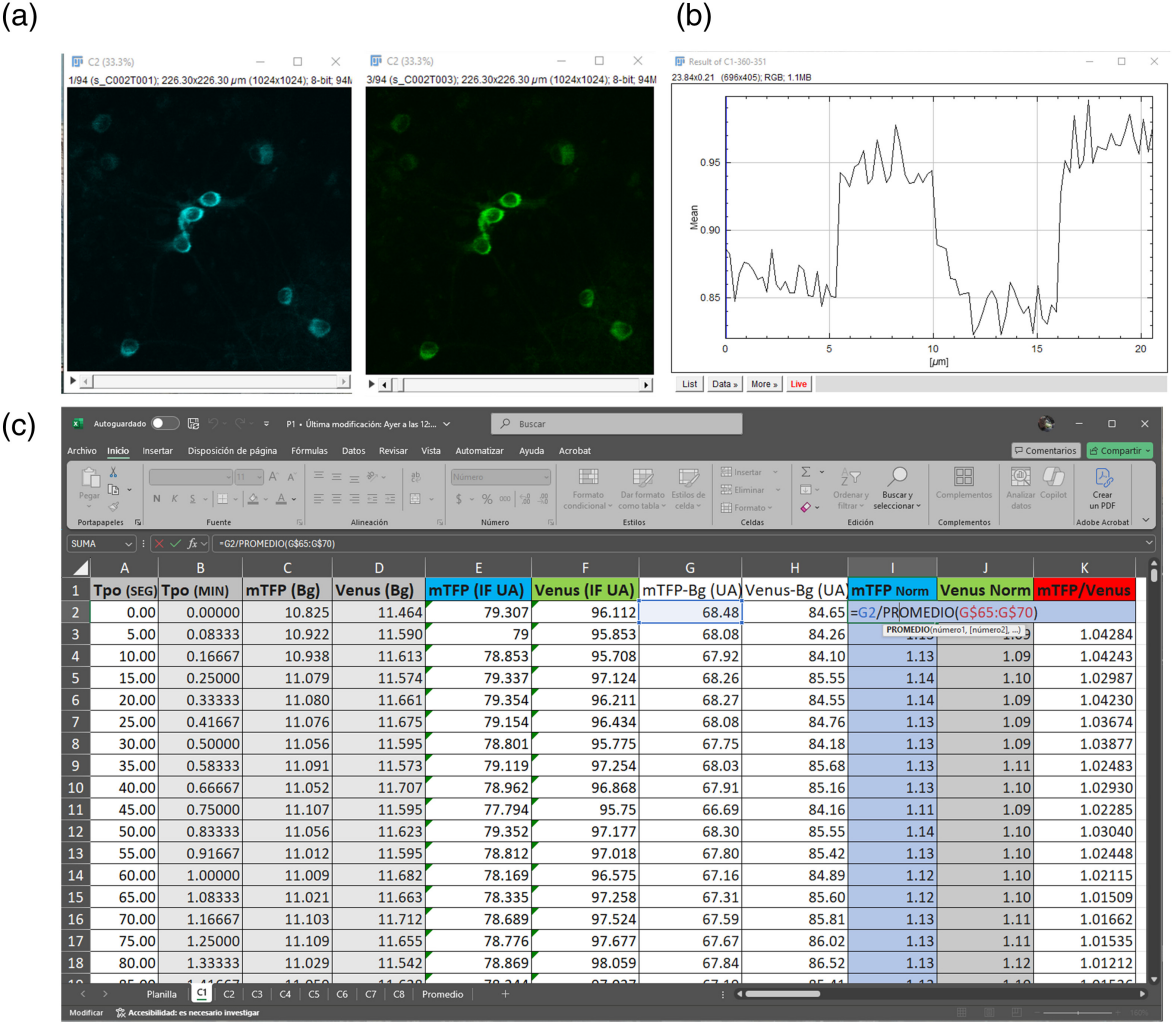
Data processing workflow for Pyronic sensor recordings. (a) Representative images of the two detection channels, mTFP (cyan) and Venus (green), are shown separately. (b) The selected cell highlights the normalization step, in which the ratio was scaled to the average value of frames within the lowest signal region. (c) A screenshot of the calculation spreadsheet. The spreadsheet includes the time in seconds and minutes, the background and cell signals from each channel separately, and the subtraction of these values.

**Tip:** Typically, we plot the background and the background-corrected mTFP and Venus fluorescence. The background should be constant unless the ROI is placed in an area where thin neurites are present, movement, or something crosses the field of view. For the mTFP and Venus fluorescence, changes in the intensity must be reciprocal between the channels, as a result of FRET. Pyronic is ratiometric, thereby insensitive to changes in cellular volume, drift, and movement. In cases of drift induced by differential and nonlinear photobleaching of the donor and/or acceptor, a cautionary note is needed. Specifically, for fuorescent indicators based on older fluorescent proteins such as CFP or YFP, drift due to photobleaching during the experiment might render the $R_{\mathrm{MIN}}$ and $R_{\mathrm{MAX}}$ non-comparable, making the two-point calibration protocol not possible without corrections. However, the use of fluorescent indicators such as Pyronic, which is based on modern fluorescent proteins such as mTFP and Venus that are resistant to photobleaching and brighter,^52^^–^^54^ allows to perform the two-point calibration protocol with comparable experimental points (Fig. 6).

**Tip:** Non-reciprocal changes must be evaluated carefully, specifically because of potential pH artefacts. The *in vitro* characterization of Pyronic indicates that the sensor is not significantly affected by pH within the physiological range.^23^ However, substantial pH changes can affect the single fluorophores to varying degrees. The pH artefact manifests as a quick change in the fluorescence of the two channels, typically in the same direction; however, the Venus channel is usually more affected. On occasions, experimental manipulation can induce a real change in the metabolic response, co-existing with a pH change; careful analysis and interpretation are advised until pH is controlled, or ruled out. Genetically encoded pH sensors and chemical dyes are available elsewhere, and controls of pH are highly recommended.

### 7.7 Spectral Bleed-through and the Two-point Calibration Method

Due to the nature of the protein pairs that comprise the FRET-based sensors, bleedthrough or signal cross-contamination between the fluorophores is inevitable. Most commonly, two sources of bleedthrough are described: first, acceptor spectral bleedthrough, and second, donor spectral bleedthrough. Acceptor bleedthrough manifests when the excitation range of the donor protein partially overlaps with the excitation range of the acceptor protein, yielding an unwanted (non-FRET) emission from the acceptor protein. Alternatively, donor spectral bleedthrough occurs when part of the emission of the donor protein is also detected, or “bleeds,” into the emission range of the acceptor protein.

**Fig. 7:**
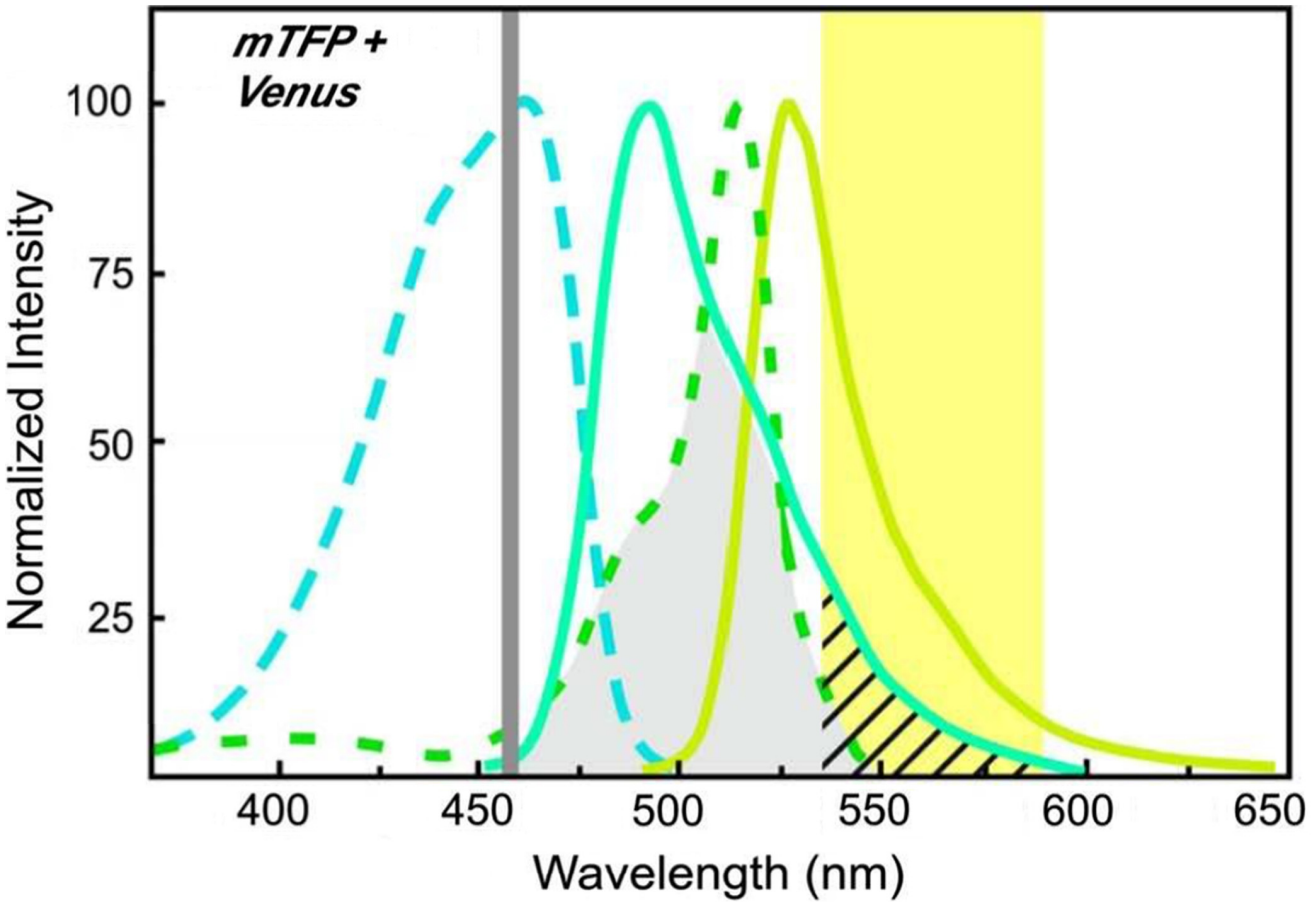
mTFP and Venus excitation and emission spectra. mTFP in combination with Venus is shown, illustrating the 458-nm laser line and the FRET emission channel (535 to 590 nm), with donor spectral bleedthrough into the FRET channel indicated by cross-hatching. This image is licensed under the Creative Commons Attribution 4.0 International License (CC BY 4.0), and it is a modified version of the original paper.^53^

In the case of the mTFP and Venus FRET-pair, acceptor bleedthrough is significantly reduced because the excitation at 458 nm coincides with the absorption maximum of mTFP (462 nm) but barely excites Venus, so the contribution of acceptor spectral bleedthrough to the FRET channel is negligible. Furthermore, most imaging setups for FRET-based imaging are equipped with excitation lines in the 420 to 440 nm range (as we also strongly recommend), ensuring proper excitation of the donor fluorophore (i.e., mTFP) while preventing cross-excitation of the acceptor fluorophore (i.e., Venus). Nonetheless, because the mTFP emission peak is shifted 17 nm toward the green relative to the CFP, and despite its emission band being narrower, there is still some important donor spectral bleedthrough in the FRET channel (Fig. 7). Under 440 nm excitation, Pyronic, as well as other FRET-based sensors comprising the mTFP-Venus pair, is almost entirely insensitive to the acceptor form of bleedthrough; however, the donor spectral bleedthrough in the FRET channel cannot be prevented.

Given that this is a common setback of FRET-based sensors, different methods to correct spectral bleedthrough have been described elsewhere.^22^^,^^55^ In brief, the donor (i.e., mTFP) or acceptor (i.e., Venus) proteins must be expressed separately. Next, the emission of the donor (mTFP) and acceptor (Venus) in the FRET emission filter is assessed in donor-protein-expressing cells upon donor excitation (440 nm). This reveals the emission of the donor (mTFP: $I_{\mathrm{mTFP}}$) and the amount of donor spectral bleedthrough, as measured by the emission collected in the FRET emission filter (mTFP bleedthrough: ${\mathrm{BT}}_{\mathrm{mTFP}}$). Finally, the emission of the acceptor (Venus) is evaluated in acceptor-protein expressing cells upon direct acceptor excitation (515 nm). This unveils the emission of the acceptor (Venus: $I_{\text{Venus}}$) and the degree of acceptor bleedthrough as the emission collected in the FRET emission filter (Venus bleed-through: ${\mathrm{BT}}_{\text{Venus}}$). Although experimentally feasible, the method is somewhat cumbersome, as it requires the expression of individual proteins in cells. Moreover, the assessment of the spectral bleedthrough varies from microscope to microscope, for it strongly depends on the optical features of the imaging setup. Therefore, it is a setup-specific measure.

Although the two-point calibration method does not explicitly correct for spectral bleedthrough, it effectively accounts for it. This is based on the observation that the primary consequence of mTFP emission bleedthrough into the Venus channel is a reduction in the apparent dynamic range of Pyronic. Such a reduction is highly dependent on the experimental setup and can significantly impact the sensor’s detection range. However, the proposed two-point calibration approach utilizes the dynamic range measured for each cell and experiment, thereby compensating for and indirectly correcting the bleedthrough effect. Therefore, when a two-point noninvasive calibration procedure is unavailable, and only a one-point calibration assessment is feasible, correction for spectral bleedthrough is mandatory.

### 7.8 Pyruvate Quantification

20. To compute the cytosolic pyruvate concentrations, the normalized Pyronic signal should be used to calculate the minimal FRET ratio $R_{\mathrm{MIN}}$ and the saturated FRET ratio $R_{\mathrm{MAX}}$. The *in vitro* Pyronic $K_{D}$, which is 107  μM, should be obtained from the original paper of Pyronic.^23^ These values should be included in the following equation:
  $$\mathrm{Pyr}\;(\mu M)=\frac{K_{D}*\Delta R}{\Delta R_{\mathrm{MAX}}-\Delta R}$$
  where $K_{D}$ corresponds to the dissociation constant obtained from an *in vitro* experiment with purified protein, ΔR is a given experimental point, and $\Delta R_{\mathrm{MAX}}$ corresponds to the experimental dynamic range obtained from each cell.

**Fig. 8:**
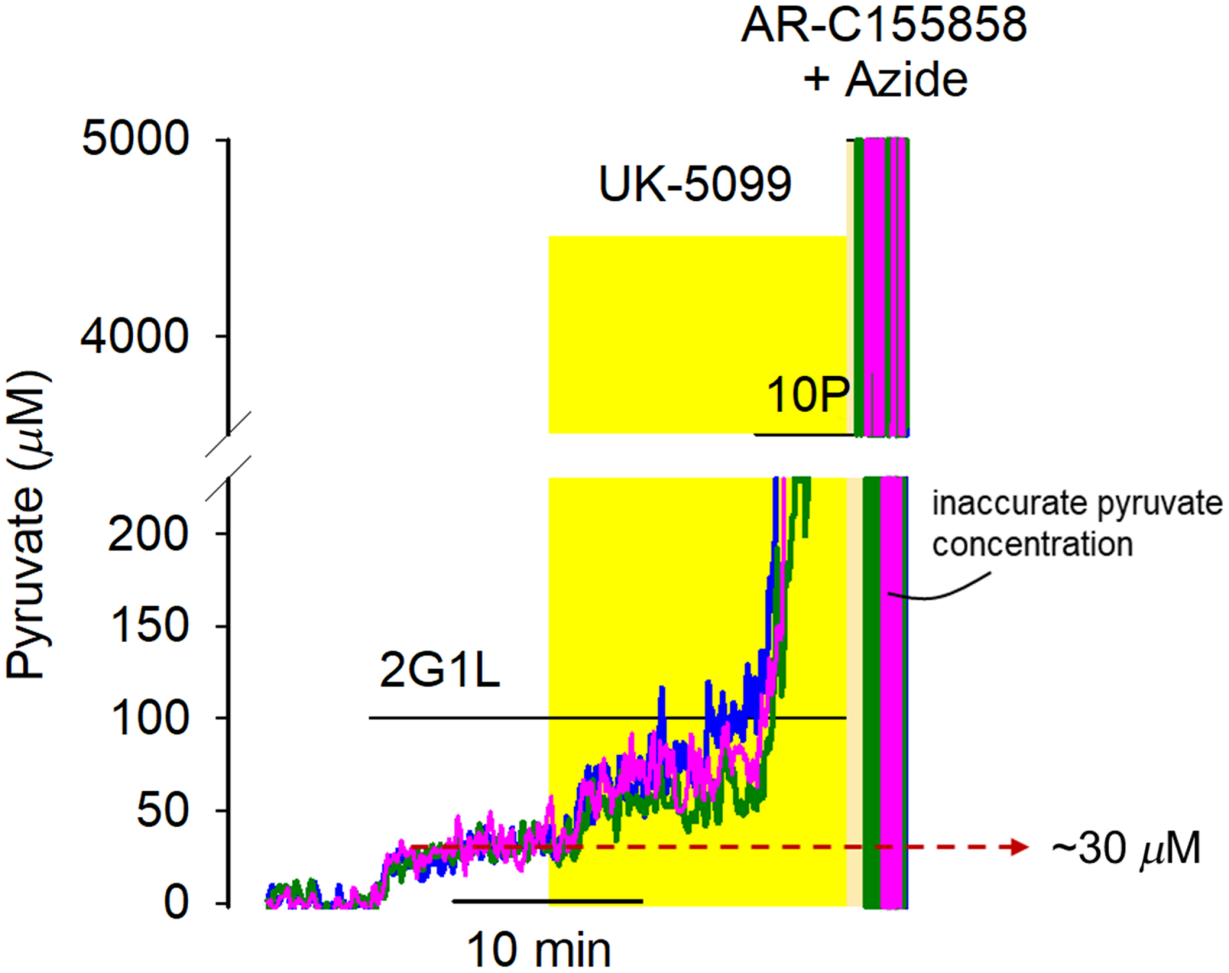
Calibrated signal of Pyronic. Cytosolic pyruvate concentration from three single hippocampal neurons obtained by the two-point calibration protocol. The reference buffer contains 2 mM glucose and 1 mM lactate (2G1L). The original data that were used to calibrate the Pyronic signal is presented in Fig. 4(b).

**Tip:** Given that the dynamic range ($\Delta R_{\mathrm{MAX}}$) is determined for each experiment, FRET ratio values significantly close to the $R_{\mathrm{MAX}}$ will result in inaccurate pyruvate concentrations. This problem arises because the calibration equation’s denominator approaches zero when the Pyronic signal becomes saturated. However, physiological cytosolic pyruvate concentrations lie in the tens of micromolar to a few hundred micromolar, a safe range where the Pyronic signal is far from saturation (Fig. 8).

## 8 Results

### 8.1 Neuronal Pyruvate Concentration

Our two-point calibration method reveals that the Pyronic signal is ∼4% above the minimum when neurons are bathed in glucose and lactate. This steady-state level likely results from the engagement of the glycolytic machinery and the backwards catalysis of LDH, which catabolizes pyruvate in the cytosol. In contrast, the pharmacological calibration raises the Pyronic signal to an ∼20%, corresponding to the maximum Pyronic dynamic range when using lentivirus as a gene-delivery system. By using the *in vitro* $K_{D}$ for Pyronic and the here-assessed $\Delta R_{\mathrm{MAX}}$, the steady-state concentration of neuronal pyruvate is ∼30  μM when neurons are supplied with 2 mM glucose and 1 mM lactate, which unveils a rather significant cytosolic pool of pyruvate in neurons.

## 9 Limitations

Certainly, two-point calibration has limitations. The introduced calibration protocol calculating cytosolic pyruvate concentration relies fundamentally on the assumed invariant $K_{D}$. However, this parameter is derived from *in vitro* experiments using purified protein in an intracellular‐mimicking buffer that approximates ionic strength but lacks the full complexity of the cellular environment. Consequently, possible discrepancies between the purified protein and the *in-cellulo* $K_{D}$ should be taken into consideration. Nonetheless, potential different $K_{D}$ will include a systematic error, making the comparison in terms of fold-change secure to be analyzed.

Pyronic’s dynamic range can be variable. Therefore, it should be correctly characterized in the cellular type of interest. The main confounding factors that affect dynamic range are different expression levels of Pyronic, because it affects the indicator’s signal-to-noise ratio. Additionally, a careful selection of MCT inhibitors to fully saturate the fluorescent indicator is mandatory. This should be made to block all the possible pyruvate exits and in this manner reach the $R_{\mathrm{MAX}}$ by intracellular pyruvate accumulation. This protocol needs to be adjusted to a specific gene delivery system and experimental samples.

Although the absolute pyruvate concentration obtained from this protocol is informative, steady-state pyruvate concentration does not tell us too much about how cell metabolism is performing. Pyronic does not distinguish between glycolytic-derived and LDH-reversed pyruvate. Therefore, coupling this protocol with pharmacological perturbation of the steady-state enables a quantitative dissection of the fluxes that shape cellular pyruvate metabolism.

## 10 Conclusion

Pyruvate is a nodal intermediate in cellular metabolism. This protocol introduced a detailed procedure for quantifying cytosolic pyruvate concentration in neurons at single-cell resolution using a minimally invasive, two-point calibration approach employing the FRET-based genetically encoded fluorescent indicator Pyronic. We envision that this protocol can also be used in other types of cells, choosing the correct MCT inhibitor, allowing a quantitative assessment of pyruvate metabolism in mammalian systems. This facilitates comparisons across different cells, samples, and experimental batches, thereby enabling comparison between a plethora of experimental conditions, which is not possible with intensity or FRET-ratio.

## Data Availability

The datasets used and/or analyzed during the current study are available from the corresponding author upon reasonable request.
